# RNA interference: learning gene knock-down from cell physiology

**DOI:** 10.1186/1479-5876-2-39

**Published:** 2004-11-22

**Authors:** Simone Mocellin, Maurizio Provenzano

**Affiliations:** 1 Department of Oncological & Surgical Sciences, University of Padova, Italy; 2 Department of Transfusion Medicine, Clinical Center, National Institutes of Health, Bethesda, MD, USA

## Abstract

Over the past decade RNA interference (RNAi) has emerged as a natural mechanism for silencing gene expression. This ancient cellular antiviral response can be exploited to allow specific inhibition of the function of any chosen target gene. RNAi is proving to be an invaluable research tool, allowing much more rapid characterization of the function of known genes. More importantly, RNAi technology considerably bolsters functional genomics to aid in the identification of novel genes involved in disease processes.

This review briefly describes the molecular principles underlying the biology of RNAi phenomenon and discuss the main technical issues regarding optimization of RNAi experimental design.

## Introduction

In 1998 Fire and coll. coined the term RNA interference (RNAi) referring to the phenomenon of post-translational silencing of gene expression that occurs in response to the introduction of double-stranded RNA (dsRNA) into a cell [1]. This phenomenon can result in highly specific suppression of gene expression. RNAi technology is rapidly spreading in research laboratories worldwide, as it is associated with a number of practical and theoretic advantages over preexisting methods of suppressing gene expression (Table 1). RNAi promises to revolutionize key areas of medical research, as demonstrated by the preliminary findings obtained in the fields of cancer, infectious diseases and neurodegenerative disorders. In this review the principles underlying this phenomenon as well as the technical challenges encountered while using RNAi for research purposes are discussed.

**Table 1:** Comparison between different methods for gene silencing.

**Method**	**Advantages**	**Drawbacks**
*RNA interference*	Specific Relatively easy	Knock-down (not knock-out) Needs transfection
*Anti-sense DNA*	Easy Inexpensive	Variable efficiency Variable specificity Needs transfection
*Dominant negative mutants*	Stable suppression Specific protein domains can be targeted	Needs transfection Variable/unexpected effect
*Knock-out animal*	Complete gene silencing	Labor intensive, expensive Lethal mutants may prevent embryonic development
*Small molecule inhibitors*	Easy delivery	Variable specificity Labor intensive development

## The physiology of RNAi

Introduction of long dsRNA into a mammalian cell triggers a vigorous nonspecific shutdown of transcription and translation, in part due to activation of dsRNA-dependent protein kinase-R (PKR) [2]. Activated PKR phosphorylates the translation initiation factor EIF2: this effect, in association with activation of Rnase-L and induction of interferon production, halts protein synthesis and promotes apoptosis. Overall, this is believed to represent an antiviral defense mechanism [3]. Owing to this phenomenon, initial observations of RNAi induced by long dsRNA in plants [4] and the nematode Caenorhabditis elegans [1] were at first applied to mammalian cells with little success. In a breakthrough experience reported by Elbashir et al., it was discovered that dsRNAs 21–23 nucleotides long – termed small interfering RNAs (siRNAs) – could suppress mammalian gene expression in a highly specific manner [5], pointing the way to gene silencing in mammalian cells.

**Figure 1:**
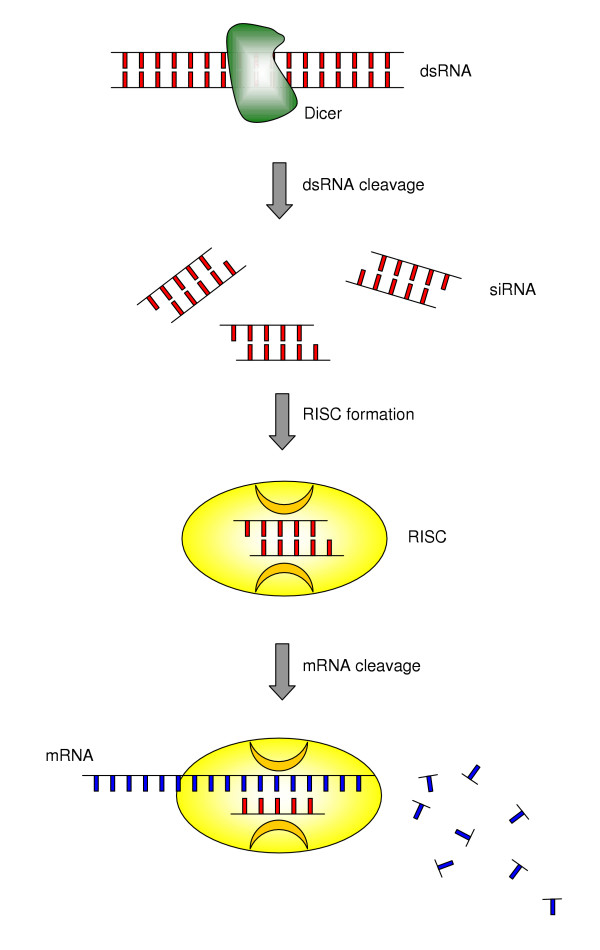
Mechanism of RNA interference (RNAi). The appearance of double stranded (ds) RNA within a cell (e.g. as a consequence of viral infection) triggers a complex response, which includes among other phenomena (e.g. interferon production and its consequences) a cascade of molecular events known as RNAi. During RNAi, the cellular enzyme Dicer binds to the dsRNA and cleaves it into short pieces of ~ 20 nucleotide pairs in length known as small interfering RNA (siRNA). These RNA pairs bind to the cellular enzyme called RNA-induced silencing complex (RISC) that uses one strand of the siRNA to bind to single stranded RNA molecules (i.e. mRNA) of complementary sequence. The nuclease activity of RISC then degrades the mRNA, thus silencing expression of the viral gene. Similarly, the genetic machinery of cells is believe to utilize RNAi to control the expression of endogenous mRNA, thus adding a new layer of post-transciptional regulation. RNAi can be exploited in the experimental settings to knock down target genes of interest with a high specific and relatively easy technology (see text for more details).

RNAi is a highly conserved mechanism throughout taxonomical species [6]. In addition to have an antiviral activity, RNAi is also believed to suppress the expression of potentially harmful segments of the genome, such as transposons, which might otherwise destabilize the genome by acting as insertional mutagens [7]. Though its mechanisms are not fully elucidated, RNAi represents the result of a multistep process (Figure 1). Upon entering the cell, long dsRNAs are first processed by the RNAse III enzyme Dicer [8]. This functional dimer contains helicase, dsRNA binding, and PAZ (named after piwi, argonaute, and zwille proteins) domains. Whereas the former two domains are important for dsRNA unwinding and mediation of protein-RNA interactions, the function of the PAZ domain species, is not completely elucidated [9,10]. Dicer produces 21–23 nucleotide dsRNA fragments with two nucleotide 3' end overhangs, i.e. siRNAs. Recently it has been suggested that Dicer has functions other than dsRNA cleavage that are required for siRNA-mediated RNAi in mammals [11]. RNAi is mediated by the RNA-induced silencing complex (RISC) which, guided by siRNA, recognizes mRNA containing a sequence homologous to the siRNA and cleaves the mRNA at a site located approximately in the middle of the homologous region [9]. Thus, gene expression is specifically inactivated at a post-transcriptional level. In C. elegans, Dicer has been shown to interact with rde proteins. The rde proteins bind to long dsRNA and are believed to present the long dsRNA to Dicer for processing [12]. Mutants displaying a high degree of resistance to RNAi have been reported to possess mutations at rde-1 and rde-4 loci [13]. Given the highly conserved nature of these enzymes, similar mutations may be of significance in mammalian cells.

Besides gene silencing, RNAi might be involved in other phenomena of gene regulation. DNA/RNA interactions are known to influence DNA methylation. It appears that RNAi can also function on this level by methylating cytosines as well as CpG sequences more classically associated with methylation. If the target sequence shares homology with a promoter, transcriptional silencing may occur via methylation. Moreover, RNA appears to interact with chromatin domains, which may ultimately direct DNA methylation. Studies of C. elegans have shown that RNAi can spread among cells through mechanisms that may not hinge upon siRNA [14]. The systemic RNA interference-deficient (sid) locus, sid-1, encodes a conserved protein with a signal peptide sequence and 11 putative transmembrane domains, suggesting that the sid-1 protein may act as a channel for long dsRNA, siRNA, or a currently undiscovered RNAi-related signal. Sid-1 mutants retain cell-autonomous RNAi but fail to show spreading of RNAi. It remains unclear whether this systemic RNAi occurs in mammals, although a strong similarity is reported between sid-1 and predicted human and mouse proteins.

## siRNA synthesis and delivery strategies

Several strategies for inducing siRNA-mediated gene silencing have been developed, each of them presenting specific advantages and disadvantages (Table 2).

**Table 2:** Comparison between siRNA delivery methods.

**Method**	**Advantages**	**Drawbacks**
*Chemical or enzymatic synthesis*	Rapid Enzymatic: no need to test individual siRNA Chemical: high purity	Transient RNAi Needs transfection Enzymatic: variable purity & specificity Chemical: expensive
*DNA plasmid vector or casette*	Less expensive Stable RNAi	Labor intensive Needs transfection
*Viral vector*	Stable RNAi May be effective in cells resistant to transfection with dsRNA/plasmids	Labor intensive Potential biohazard

Synthesis, purification, and annealing of siRNAs by industrial chemical processes [15] is becoming increasingly popular. This method is rapid and purity is generally high. This may be the best approach for initial "proof of principle" experiments. In vitro siRNA synthesis is an alternative and relies upon the T7-phage polymerase [16]. This polymerase produces individual siRNA sense and antisense strands that – once annealed – form siRNAs. Extra nucleotides required by the T7 promoter are removed by RNase digestion and cleaning steps. Otherwise, recombinant Rnase-III can be used to cleave long dsRNAs to produce multiple siRNAs [17]. Although technically easy, this approach presents the drawback of the generation of non-specific siRNAs. siRNAs can be produced by polymerase-III promoter-based DNA plasmids or expression cassettes [18]. These constructs produce small inverted repeats, separated by a spacer of three to nine nucleotides, termed short hairpin RNAs (shRNAs), which are processed by Dicer into siRNAs [19]. Transcription begins at a specific initiation sequence, determined by the promoter used. In addition to a defined initiation sequence, the U6 polymerase-III promoter terminates with TTTT or TTTTT [20]. The products are shRNAs that contain a series of uridines at the 3' end, a feature that seems to favor RNAi [21].

Suppression of gene expression by RNAi is generally a transient phenomenon [22]. Gene expression usually recovers after 96 to 120 hours or 3 to 5 cell divisions after transfection, which is likely due to dilution rather than degradation of siRNAs. However, by introducing plasmids which express siRNA and a selection gene, stable RNAi can be sustained as long as two months after transfection [23]. Interest is growing in the use of viral vector-mediated RNAi. Adenoviral and retroviral vectors have been reported to produce siRNAs in vivo [24,25] and stable RNAi is obtained using this method, though in the absence of a selective pressure [26,27]. Virus-mediated RNAi may circumvent some of the problems associated with cells that are generally refractory to RNAi, such as non-transformed primary cells [28]. At present, the question of whether functional RNAi will continue in all progeny of a cell with stable vector integration remains unanswered.

## Designing RNAi experiments

Several crucial considerations should be beard in mind while designing RNAi experiments. The below examples regard RNAi experiments performed with chemically synthesized siRNA.

1. The first step is to design a suitable siRNA sequence. A growing number of libraries of validated siRNAs directed toward some frequently targeted genes are available. However, if the gene of interest has not been targeted using siRNA before, a novel siRNA must be developed. In mammalian cells RNAi is mediated by 21- to 23-nucleotide siRNAs containing symmetrical two nucleotide 3' overhangs. Given a siRNA sequence alone, it is not currently possible to predict the degree of gene knockdown produced by a particular siRNA. Nevertheless, several observations have been made that can be taken into account to increase the probability of producing an effective siRNA. The chief variable is the gene target site. Generally, it is recommended that a target site located at least 100–200 nucleotides from the AUG initiation codon is chosen. Targets within 50–100 nucleotides of the termination codon should instead be avoided. The 5' and 3' untranslated region (UTR) should also be avoided, since associated regulatory proteins might compromise RNAi. This is just a general recommendation, as some siRNAs targeting the 3' UTR have also been shown to induce RNAi [29]. Numerous on-line design tools will produce a list of suitable gene target sites. It is important to ensure that the sequence is specific to the target gene by performing a BLAST search in order to avoid cross reaction with unwanted genes. As an example, Biocomputing at the Whitehead Institute for Biomedical Research – a nonprofit independent research and educational institution affiliated with the Massachusetts Institute of Technology – is one of several organizations that has developed a freely available web-based siRNA design tool.

2. The structural characteristics of the siRNA molecules are another crucial aspect to be considered while designing RNAi experiments. SiRNA of 21 nucleotides with 3'-d(TT) or (UU) overhangs are considered the most effective [30]. Despite the fact that nucleotide-protein steric interactions contribute to the relationship between siRNA length and activity, the reason for this relationship is not completely elucidated. For optimal siRNA secondary structure, the GC ratio should ideally be between 45 and 55%, and multiple identical nucleotides in series, particularly poly(C) and poly(G), should be avoided to determine any requirements for modification, such as fluorophore labeling to allow for siRNA tracking and quantification of transfection efficiency.

3. To induce RNAi, siRNA must be transfected into the cells of interest. Several transfection reagents exist, the most commonly used being liposomal or amine-based. In some cases electroporation may be used, but cell toxicity can be high with this technique [31]. Cell lines show varying responses to different transfection reagents, and it may be necessary to try more than one reagent or approach. Transfection efficiency is optimized by titrating cell density, transfection time, and the ratio of siRNA-to-transfection reagent. The cell passage number and antibiotic use can also affect the efficiency of transfection.

4. Recently, experimental design features have been suggested to guarantee the rigor of RNAi experiments [32]. Due to the high specificity of RNAi, a siRNA with a one-nucleotide sequence mismatch can serve as a negative control. If this approach is used, absence of homology with other targets should be confirmed at the design stage. It is important to remember that mismatched siRNAs could target mutant gene sequences. Therefore loss of functional target gene silencing should be demonstrated to validate this approach. Alternatives include sequences that preset no homology to any known gene. Some investigators have suggested that scrambled siRNA is not sufficiently homologous to the target sequence to function as an adequate control; therefore, they propose a combination of mismatched and scrambled controls [32]. A more challenging functional control is to demonstrate the "rescue" of the target gene function following artificial overexpression of the target gene. Transfection of a plasmid expressing the gene sequence to which a siRNA is targeted results in production of mRNA that would also be targeted by the siRNA. This problem can be overcome using plasmids containing silent mutations. This approach takes advantage of degeneracy of gene coding, i.e., amino acids are represented by more than one three-nucleotide codon sequence. Rescue is achieved by expression of a protein identical to the native protein from a nucleotide sequence that differs from the native nucleotide sequence to which the siRNA is targeted. Alternatively, siRNAs directed to the 3'-UTR can be used. Many researchers use more than one siRNA, with each targeted to different areas of the gene sequence. A consistent RNAi response using different siRNAs with a variety of targets within the gene sequence of interest would increase confidence in experimental results. Dose-respons characteristics should be determined and the lowest effective concentration of siRNA be used to avoid nonspecific effects.

5. The effect of RNAi should be quantified at both the mRNA and the protein level. The knockdown of a protein should be probably evaluated after mRNA reduction has been proved: in fact, a reduction in protein levels not accompanied by a decrease in mRNA might indicate that other mechanisms are at work, such as RNAi mediated by microRNA. Northern blot analysis is considered by many to be the gold standard. Real-time reverse transcriptase polymerase chain reaction, incorporating internal controls to quantify "housekeeping" gene transcript levels, can also be used [33]. Protein knockdown can be confirmed by Western blot analysis, immunofluorescence, flow cytometry and phenotypic and/or functional assays. Although RNAi generally occurs within 24 h of transfection, both onset and duration of RNAi depend on the turnover rate of the protein of interest, as well as the rate of dilution and longevity of the siRNAs. The duration of gene silencing can also be modified by factors such as the concentration of serum in the culture medium, which affects cell-cycle rate. It is therefore necessary to determine the time course of any silencing observed under specific conditions using the modalities discussed.

## Conclusions

RNAi is now commonly used in biological and biomedical research to study the effect of blocking expression of a given gene. As the effect is rarely complete, it is generally termed a "knock-down" to distinguish it from the "knock-out" achieved by deletion of the gene. Although significant advances have been made as compared to previous methods, RNAi has its own limitations. Not every sequence works, most researchers reporting a success rate of about one in three. Moreover, although the effects are generally believed to be highly sequence-specific, some doubts remain as to whether or not some of the observed effects are "off target." Some residual activation of the interferon system has been reported, as well as degradation of closely related, but non-identical, mRNAs. Nevertheless, RNAi remains the most promising functional genomics tool recently developed. DNA microarray technology has now enabled the level of expression of every gene in the genome to be determined under any condition [34,35]. This has led to a huge accumulation of data on genes whose expression is significantly altered in several diseases. To take an example, large databases have been established of genes that are pathologically regulated in cancer. In some cases this has resulted in the identification of key genes involved in tumor development and provided important new therapeutic targets. However, in most cases the pattern of gene expression is far too complex to allow for identification of the relatively small number of misexpressed genes that are involved in causing or maintaining the disease rather than the much larger number that are innocent bystanders. The ability of RNAi to provide relatively easy ablation of gene expression has opened up the possibility of using collections of siRNAs to analyse the significance of hundreds or thousands of different genes whose expression is known to be upregulated in a disease, given an appropriate tissue culture model of that disease. Perhaps more important still is the possibility of using genome-wide collections of siRNAs, whether synthetic or in viral vectors, as screening tools. Two main avenues of research can rely upon RNAi libraries. First, in a high-throughput manner each gene in the genome is knocked-down one at a time and the cells or organism scored for a desired outcome, such as death of a cultured cancer cell but not a normal cell. Due to the very large numbers of assays required to screen all 35–50,000 genes in the human genome, this approach is highly labor-intensive and time consuming. The other approach is to use large pools of RNAi viral vectors and apply a selective pressure that only cells with the desired change in behavior can survive. The identity of the genes knocked-down in the surviving cells can then be identified by sequencing the RNA interference vectors that they carry. Both approaches show consider-able promise in identifying novel genes that may make important therapeutic targets for inhibition either by conventional drug discovery methods or, more intriguingly, by RNA interference itself.
